# Melt Blown Fiber-Assisted Solvent-Free Device Fabrication at Low-Temperature

**DOI:** 10.3390/mi11121091

**Published:** 2020-12-10

**Authors:** Minjong Lee, Joohoon Kang, Young Tack Lee

**Affiliations:** 1 Department of Electrical and Computer Engineering, Inha University, Incheon 22212, Korea; ydr8273@inha.edu; 2 School of Advanced Materials Science and Engineering, Sungkyunkwan University (SKKU), Suwon 16419, Korea

**Keywords:** solvent-free lithography, transition metal dichalcogenides, melt blown fiber, micro-scaled shadow mask, complementary metal-oxide-semiconductor

## Abstract

In this paper, we propose a solvent-free device fabrication method using a melt-blown (MB) fiber to minimize potential chemical and thermal damages to transition-metal-dichalcogenides (TMDCs)-based semiconductor channel. The fabrication process is composed of three steps; (1) MB fibers alignment as a shadow mask, (2) metal deposition, and (3) lifting-up MB fibers. The resulting WSe_2_-based p-type metal-oxide-semiconductor (PMOS) device shows an ON/OFF current ratio of ~2 × 10^5^ (ON current of ~−40 µA) and a remarkable linear hole mobility of ~205 cm^2^/V·s at a drain voltage of −0.1 V. These results can be a strong evidence supporting that this MB fiber-assisted device fabrication can effectively suppress materials damage by minimizing chemical and thermal exposures. Followed by an MoS_2_-based n-type MOS (NMOS) device demonstration, a complementary MOS (CMOS) inverter circuit application was successfully implemented, consisted of an MoS_2_ NMOS and a WSe_2_ PMOS as a load and a driver transistor, respectively. This MB fiber-based device fabrication can be a promising method for future electronics based on chemically reactive or thermally vulnerable materials.

## 1. Introduction

Microfabrication techniques have been persistently developed as industrial standards increase for high-performance next-generation electronic device applications. Photolithography has been a commonly used method for the spatially precise patterning process in the integrated circuit (IC) technology. Although this conventional photolithography can provide high-resolution patterns, process complexity and high-cost equipment should be involved. Furthermore, several steps incorporated with solvent and thermal exposure, such as photoresist (PR) coating, curing, and developing, can cause detrimental effects to fully exploit intrinsic properties of semiconductors. In order to overcome the limitations, soft lithography has been considered, as promising alternatives have many advantages, such as low-cost, low-temperature processable, and chemical-free methods [1,2]. Although the PR-based patterning steps are not involved during the soft-lithography process, which can avoid chemical and thermal exposure, poor electrical contacts between metal-semiconductor junctions are still remaining as a challenge. Beyond the electronic device fabrications, chemical and thermal exposure-free approaches are desired in the field of display. To handle chemically sensitive organic materials for organic-light-emitting diode fabrication, a chemical-free fabrication process is highly demanded to minimize potential chemical reaction of organic materials. In an effort to find solutions to overcome such limitations, several approaches have been introduced, including inkjet printing [3], fine-metal-shadow masking [4,5], and microcontact-printing [6].

In a decade, two-dimensional (2D) van der Waals semiconductors such as transition-metal-dichalcogenides (TMDCs) have been intensively studied to fully exploit their superlative electronic properties [7,8,9,10]. For example, MoS_2_ and WSe_2_ as semiconducting members of TMDCs have been extensively studied with great possibilities for future electronics within high-speed [11], flexible [12], and immune short channel effects in the device, which scale down [13]. Similar to the other semiconductors, however, the photolithography process also causes electrical property degradations of such semiconductors, due to solvent-induced chemical reactions and/or thermal degradation [14,15]. Furthermore, securing a clean interface between the source/drain (S/D) electrodes and a TMDCs channel layer is another significant issue towards high-performance device demonstrations [16,17,18,19]. Here, we report a melt blown (MB) fiber-assisted solvent-free lithography method for fabrication of field-effect transistors (FETs). The resulting electrical behaviors of TMDCs-based FETs are thoroughly compared with those of devices fabricated using a conventional photolithography process.

## 2. Materials and Methods

TMDCs-based FETs were fabricated on a thermally oxidized 285 nm-thick SiO_2_/p^+^ silicon substrate. Silicon substrate was ultrasonically cleaned by sequentially immersing in acetone, methyl alcohol, and isopropyl alcohol each for 15 min. Polydimethylsiloxane (PDMS) stamps were used to exfoliate and transfer 2D semiconductor active channels to a designated place. To fabricate PDMS stamps, base resin and cross-linker (Sylgard 184, Dow Corning) solutions were mixed with a 10:1 volume ratio, and trapped-air bubbles were removed by degassing in a vacuum for 20 min. The solution was then poured onto a cleaned silicon wafer mold and thermally cured on a hot plate in ambient condition at 423 K for 1 h.

**Figure 1:**
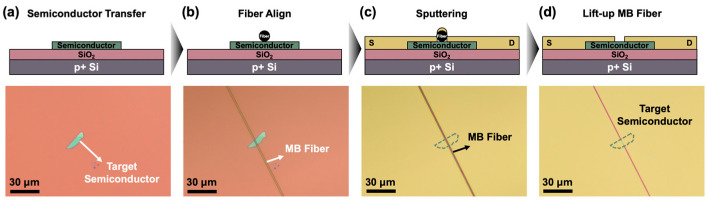
The non-lithographic micro-scaled device fabrication flow steps; (**a**) 2D transition-metal-dichalcogenides (TMDC) transferring on the silicon substrate; (**b**) melt-blown (MB) fiber alignment on the target TMDC nanoflake; (**c**) metal sputtering; (**d**) lifting-up MB fiber for S/D electrodes formation.

Figure 1 shows a non-lithographic micro-scaled device fabrication process using an MB fiber-based shadow mask. First of all, 2D TMDC semiconductors were mechanically exfoliated and transferred on a silicon substrate by a PDMS-based direct imprinting method, as shown in Figure 1a. In Figure 1b, an MB fiber-based shadow mask was aligned and subsequently transferred onto a targeted TMDC nanoflake under an optical microscope (OM) to define micropatterned S/D electrodes. The MB fiber-based shadow mask consisted of a punched PDMS frame and a selected MB fiber with an average diameter of ~1.5 µm. The suspending MB fiber was attached on the bottom side of the punched PDMS stamp (see Figure S1 with further details of MB fiber-based shadow mask technique in the Supplementary Materials (SM) section). The MB fiber could be efficiently attached at the 2D TMDC semiconductors on a silicon substrate due to its strong electrostatic force. Figure 1c,d shows a metal S/D electrodes patterning using a DC magnetron sputtering system, and we named this process as the “lift-up” method. By lifting-up the MB fiber-based shadow mask, a narrow gap between S/D electrodes (channel length) was formed with a distance corresponding to the diameter of a used MB fiber. This method is a straightforward way to form micro-scaled S/D electrodes without PR-casting and thermal curing process, and it can effectively reduce the whole process steps and the cost compared to the conventional photolithography. Based on this approach, WSe_2_ and the MoS_2_-based PMOS and NMOS devices were fabricated with Pt (50 nm) and Ti/Au (25 nm/25 nm) as S/D electrodes, respectively.

## 3. Results and Discussion

**Figure 2:**
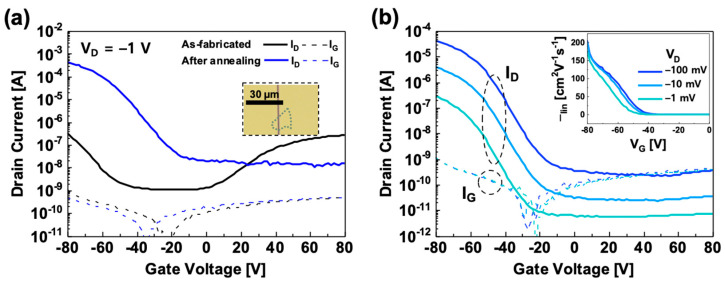
(**a**) Transfer characteristic curves of the WSe_2_ p-type metal-oxide-semiconductor (PMOS) before (as-fabricated) and after the post-annealing process at *V_D_* of −1 V (W/L = 10); (**b**) Transfer characteristic curves and linear mobility plot (inset) of the post-annealed WSe_2_ PMOS at *V_D_* of −1 mV, −10 mV, and −100 mV.

Figure 2 shows drain current-gate voltage (*I_D_*-*V_G_*) transfer characteristic curves of a WSe_2_-based PMOS transistor. As shown in Figure 2a, the as-fabricated WSe_2_ PMOS shows an ambipolar behavior, and both drain ON current (*I_ON_*) levels in p-type and n-type regions (*I_ON,p_* and *I_ON,n_*) were ~0.3 µA at a drain voltage (*V_D_*) of −1 V (black line). After a post-annealing process (423 K for 1 h in ambient air), the WSe_2_ PMOS shows a strong p-type property, while the n-type characteristic is suppressed (blue line). The thermally annealed WSe_2_ PMOS shows excellent *I_ON,p_* of ~−0.4 mA at *V_D_* of −1 V, which is ~10^3^ times higher *I_ON,p_* than the as-fabricated device at the same bias conditions. It implies that the post-annealing process in an ambient condition forms an atomically thin tungsten oxides (*WO_x_*) layer, having a p-type electrical characteristic on the surface of WSe_2_ nanoflake, and it can be understood as a p-doping process at the WSe_2_ channel surface [20,21,22,23]. Figure 2b shows *I_D_*-*V_G_* transfer characteristic curves and linear mobility (*µ_lin_*) plot (inset) at *V_D_* of −1 mV, −10 mV, and −100 mV. The *µ_lin_* was calculated by using the following equation:

$$\mu_{lin}=\left(\frac{dI_{D}}{dV_{G}}\right)\left(\frac{L}{WC_{OX}\left|V_{D}\right|}\right)$$

where, *C_OX_* is the capacitance of SiO_2_ gate insulator, W and L are the width and the length of the FET channel, respectively. From this equation, the maximum linear mobility (*µ_lin,max_*) of our WSe_2_ PMOS was calculated as ~205 cm^2^/V·s at *V_D_* of −100 mV. Moreover, another WSe_2_ PMOS also showed excellent *µ_lin,max_* of ~244 cm^2^/V·s, as shown in Figure S2 in Supplementary Materials. As shown in Figure 2b, the *I_D_* of our WSe_2_ PMOS is proportionally increased by the *V_D_* variation. This result can be a strong evidence that our non-lithographic fabrication method provides a high-quality Ohmic contact between the WSe_2_ and Pt S/D electrodes. As a result, we can successfully achieve the high-performance WSe_2_ PMOS device with excellent *µ_lin,max_*.

**Figure 3:**
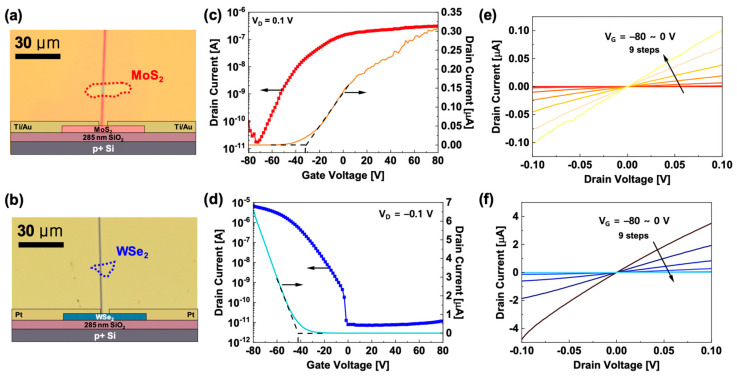
(**a**,**b**) Device schematic structures and OM images of the MoS_2_ n-type metal-oxide-semiconductor (NMOS) and the WSe_2_ PMOS; (**c**,**d**) Transfer characteristic curves of the MoS_2_ NMOS and the WSe_2_ PMOS; (**e**,**f**) Output characteristic curves of the MoS_2_ NMOS and the WSe_2_ PMOS, respectively.

In addition to the WSe_2_-based PMOS device, an n-type MoS_2_ nanoflake-based NMOS device was investigated in a similar manner. Figure 3a,b shows schematics of device structures and OM images of an MoS_2_ NMOS and an annealed WSe_2_ PMOS on 285 nm-thick SiO_2_/p^+^ silicon substrate, respectively. Figure 3c,d show *I_D_*-*V_G_* transfer characteristics of each device. The scattered symbol and solid line display the semi-logarithmic and linear *I_D_*, respectively. The MoS_2_ NMOS shows *I_ON_*/*I_OFF_* ratio of ~2 × 10^4^, a threshold voltage (*V_th_*) of ~−32.6 V, and *I_ON_* of ~0.3 µA at *V_D_* = 0.1 V, while the WSe_2_ PMOS shows higher *I_ON_/I_OFF_* ratio of ~10^6^, *V_th_* of ~−42.1 V and higher *I_ON_* of ~−6.5 µA at *V_D_* = −0.1 V. Figure 3e,f show the *I_D_*-*V_D_* output characteristic curves of the MoS_2_ NMOS and WSe_2_ PMOS for a *V_G_* range of −80 V to 0 V with +10 V step increment. Both output curves clearly show excellent ohmic contact behaviors of the MoS_2_/Ti-Au contact and the WSe_2_/Pt contact within the linear operation region.

**Figure 4:**
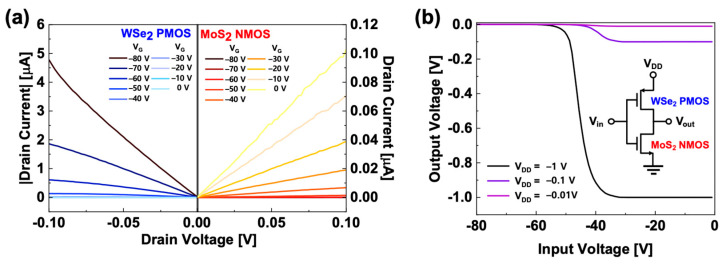
(**a**) Output characteristics curves of the WSe_2_ field-effect transistors (FET) (driver) and MoS_2_ FET (load) for complementary metal-oxide-semiconductor (CMOS) inverter circuit application; (**b**) VTC curves of our CMOS inverter circuit device at *V_DD_* of −0.01 V, −0.1 V, and −1 V. The inset is the circuit diagram.

Based on the PMOS and NMOS devices, a CMOS inverter circuit application was implemented, and *I_D_*-*V_D_* output characteristics curves of PMOS and NMOS FETs are displayed in Figure 4a (see Figure S3 with further details of load-line analysis in the Supplementary Materials section). The MoS_2_ NMOS and WSe_2_ PMOS were used as a load and a driver transistor, respectively, because the MoS_2_ NMOS and WSe_2_ PMOS showed negative *V_th_*. Moreover, the WSe_2_ PMOS had better electrical performances, such as higher *I_D_* and linear hole mobility, than those of the MoS_2_ NMOS device. Figure 4b shows the voltage transfer characteristic (VTC) curves of our CMOS inverter circuit device, and the transition voltage is ~−42 V, which is well-matched with *V_th_* of WSe_2_ PMOS driver transistor. The inset of Figure 4b shows our CMOS inverter circuit diagram by connecting with the MoS_2_ NMOS and WSe_2_ PMOS through an Au wire-bonding technique. To the best of our knowledge, this is the first demonstration of a 2D nanomaterial-based CMOS inverter circuit application through fully dried fabrication processes.

## 4. Conclusions

We demonstrated a solvent-free device fabrication method using an MB fiber-based shadow mask to minimize high-temperature and/or solvent-induced chemical degradation of semiconducting materials. This process effectively reduces the entire process protocols and the cost compared to the conventional photolithography. Moreover, the pattern size is easily tunable based on the diameter of the MB fiber (~1.5 µm). As a result, our WSe_2_ PMOS shows excellent electrical performances, such as *µ_lin,max_* of 205 cm^2^/V·s, *I_ON_* of ~−40 µA, and *I_ON_/I_OFF_* ratio of ~2 × 10^5^ at *V_D_* = −0.1 V, because this approach provides a high-quality interface between the semiconductor active channel and the metal S/D electrode. Lastly, we successfully achieved the CMOS inverter circuit demonstrations consisted of a WSe_2_ PMOS as a driver and an MoS_2_ NMOS as a load transistor. This micro-scaled shadow masking process exhibits a promising key technique to unlock the unlimited potential of materials for future advanced electronics.

## Supplementary Materials

The following are available online at https://www.mdpi.com/2072-666X/11/12/1091/s1, Figure S1: MB fiber-based shadow masking technique, Figure S2: Transfer characteristic curves and linear mobility plot of another WSe_2_ PMOS, Figure S3: The load-line analysis for CMOS inverter circuit.
