# Supplementary material for: Melt Blown Fiber-Assisted Solvent-Free Device Fabrication at Low-Temperature
Source: Micromachines (Basel). 2020 Dec 10;11(12):1091. doi: 10.3390/mi11121091 (PMC7763187; doi:10.3390/mi11121091)
Supplement: Supplementary file 1 [file micromachines-11-01091-s001.pdf]

Supplementary Materials: Melt Blown Fiber-Assisted Solvent-Free Device Fabrication at Low-Temperature

Minjong Lee, Joohoon Kang and Young Tack Lee

Experimental Information

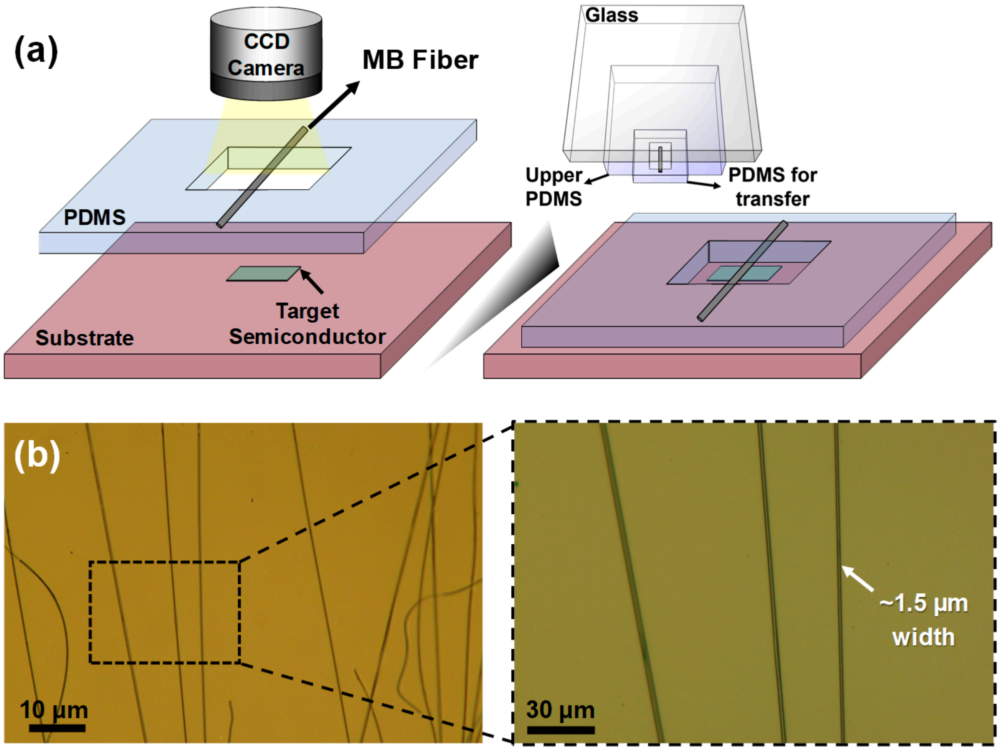

Figure S1. (**a**) MB fiber-based shadow masking technique which aligned under the OM system and subsequently attaching on the target semiconductor channel; (**b**) OM images of the MB fiber. The MB was extracted from a commercial MB filter (MB filter, Yu Han Greentech).

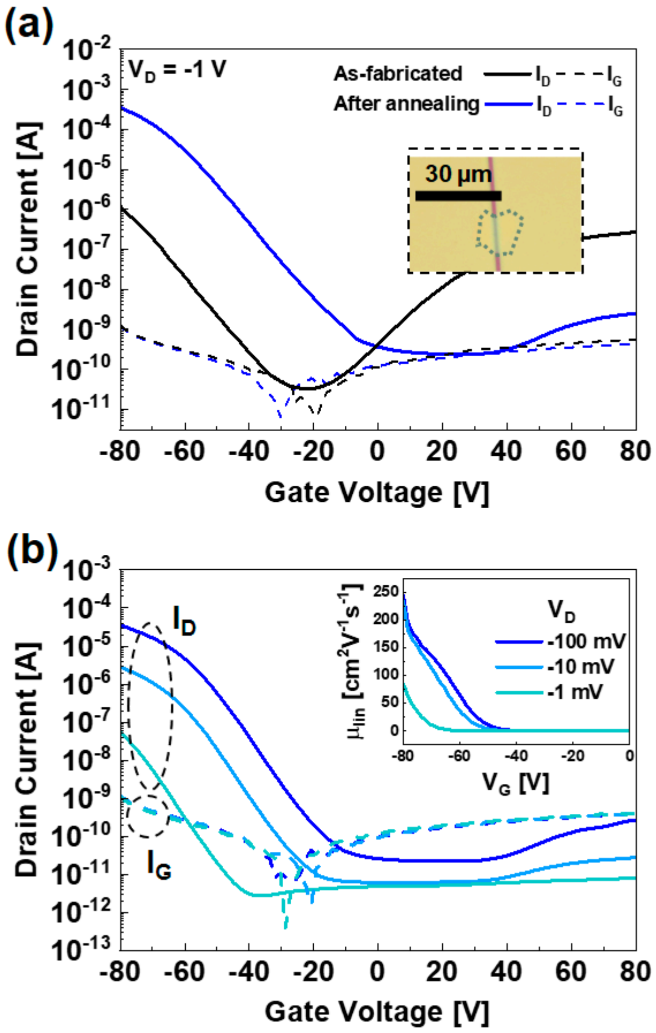

Figure S2. (**a**) Transfer characteristic curves of the WSe_2 PMOS before (as-fabricated) and after the post-annealing process at VD of −1 V (W/L = 9); (**b**) Transfer characteristic curves and linear mobility plot (inset) of the post-annealed WSe2 PMOS at VD of −1 mV, −10 mV, and −100 mV.

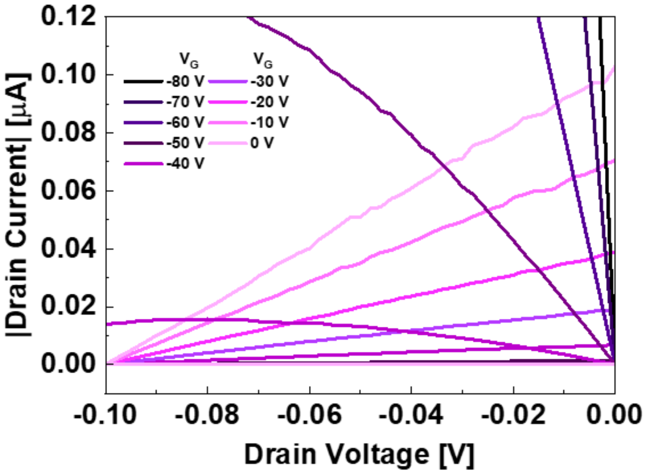

Figure S3. The load-line analysis based on MoS_2 NMOS and WSe_2 PMOS for CMOS inverter circuit.
